# Towards optimized CT lung cancer screening scan protocols

**DOI:** 10.1093/bjr/tqag066

**Published:** 2026-03-26

**Authors:** Gareth R Iball, Charlotte A Porter

**Affiliations:** Faculty of Health and Social Care, University of Bradford, Bradford, West Yorkshire, BD7 1DP, United Kingdom; Department of Medical Physics and Engineering, Leeds Teaching Hospitals NHS Trust, Leeds, West Yorkshire, LS1 3EX, United Kingdom

**Keywords:** computed tomography, lung cancer, screening, scan protocols, radiation dose, image quality, lung nodules

## Abstract

**Objectives:**

To evaluate radiation dose and image quality for CT lung cancer screening protocols and to determine which reconstruction kernels deliver accurate nodule volumetry.

**Methods:**

A variable size anthropomorphic chest phantom and a lung screening image quality phantom were scanned on 17 different scanner models. Dose metrics from the chest phantom scans were compared against international dose recommendations. Image quality phantom scans were used to assess whether the protocol yielded accurate nodule volumetry as per the Quantitative Imaging Biomarkers Alliance (QIBA) criteria, and to determine whether changes were needed to enable this.

**Results:**

Doses varied by up to a factor of 7 for the largest phantom size. All protocols delivered doses below the UK standards but only 2 scanners met the European dose criteria. Many scanners failed to meet the QIBA criteria with lung reconstruction kernels, primarily due to excessive edge enhancement and insufficient 3D spatial resolution. With alternative reconstruction kernels, the QIBA criteria were met on all bar 2 scanners.

**Conclusions:**

Standard lung screening CT protocols on contemporary scanners produce images of varying image quality, over a wide range of radiation doses. Scanner-specific reconstruction kernel selection is required to meet the QIBA criteria. The study findings enable the development of optimized CT scanning protocols for lung cancer screening program.

**Advances in knowledge:**

Standard lung cancer screening scanning protocols demonstrate notable variations in dose. Lung reconstruction kernels often yield inaccurate measurements of nodule volume; instead, soft tissue kernels are necessary to obtain reliable results.

## Introduction

The use of CT scanning for lung cancer screening (LCS) has been the subject of many research trials and pilot studies over the past 2 decades.^1–9^ Following several research trials within the UK, the Targeted Lung Health Check programme was piloted in 2019, with the aim of this being implemented across all areas of England from 2024.^10^ More recently the UK National Screening Committee has recommended that “the 4 [UK] nations move towards implementation of targeted lung cancer screening with integrated smoking cessation service provision.”^11^

Within LCS trials, a wide range of CT scanners, scan protocols, and image reconstruction settings have been used. It is widely known that scan protocol settings significantly affect the radiation doses delivered to patients.^12^ Similarly, image reconstruction settings, such as the image slice thickness, slice increment, reconstruction kernel and use of iterative reconstruction or deep learning algorithms affect the accuracy of lung nodule volumetry measurements.^13–16^ Whilst existing LCS protocols routinely use thin image slices (≤1.25 mm) and overlapping reconstructions,^17^ there is a lack of guidance on the reconstruction kernels that should be used to ensure accurate nodule sizing. Accurate nodule volumetry is of particular importance when using images from subsequent screening rounds to calculate nodule volume doubling time as this is used as a predictor of malignancy.^18–20^

Within screening scenarios the benefits of the screening test must significantly outweigh any risks.^21^ Optimized CT scanning protocols are required to generate high-quality images that enable accurate volumetry at low dose. Optimization of all scanners used in a LCS program will ensure a balance of benefit and risk is reached, whilst also securing consistency of objectively measured image quality. Technical requirements for LCS scans are available from a number of sources and whilst these provide recommendations around radiation dose levels,^10^^,^^22^^,^^23^ specific image quality requirements are only rarely mentioned.^24^ However, the Quantitative Imaging Biomarkers Alliance (QIBA) small lung nodule profile (SLN profile) provides quantitative image quality criteria that need to be met to ensure that consistently accurate nodule volumetry is obtained.^25^ This profile specifically relates to solid, non-calcified nodules, with diameters in the range of 5-12 mm. These criteria could be adopted into LCS programs as specific image quality standards.

The 6 image quality criteria are:

- Edge enhancement—shall not exceed 5%
- Spatial warping—spatial warping of less than 0.3 mm root mean square error
- 3D resolution—the standard deviation of the 3D Point Spread Function (PSF) ellipsoid volume of less than or equal to 1.5 mm^3^
- 3D resolution aspect ratio—a Z-axis PSF sigma less than 2× larger than the in-plane PSF sigma
- Hounsfield Unit (HU) bias—HU value deviation of less than 35 HU for Air and Acrylic
- Noise—a standard deviation that is ≤50 HU for homogeneous Air and Acrylic

A suitable scan protocol should meet these criteria up to a radial distance of 160 mm from the isocenter as this represents the greatest extent of lung tissue from isocenter for a well-positioned patient.^26^ Complying with the QIBA SLN profile specifications and requirements ensures accurate nodule volumetry and that the calculation of nodule volume change is within the 95% confidence interval.

Whilst CT manufacturers routinely provide a “low dose lung” scanning protocol on modern CT systems, there is minimal comparative data which allow scan protocols to be directly compared. One protocol repository is provided by the American Association of Physicists in Medicine (AAPM).^27^ Within their protocol library the AAPM state: “The Lung Cancer Screening Protocols described in this document are a set of reasonable protocols developed by the AAPM’s Working Group on Standardization of CT Nomenclature and Protocols that are to be used in the specific context of Lung Cancer Screening. These protocols were based in part on manufacturers’ Low Dose Chest protocols but were adapted based on the Working Group’s experience with the National Lung Screening Trial and other screening studies.” The scan protocol for the Yorkshire Lung Screening Trial (YLST)^28^ was based on the suggested AAPM protocol with adjustments to ensure that the requirements of the QIBA SLN profile^25^ were met.

Undertaking optimization across a screening program is challenging due to difficulties in being able to objectively assess both dose and image quality across a range of scanners, especially where the screening program is spread over a large geographical region, as was the case in both NLST and NELSON.^1^^,^^2^ Ideally those undertaking such optimization would need access to all scanners used for LCS and have a suitable range of dose and image quality test equipment. This should include a variable size anthropomorphic phantom, for assessment of patient dose, and an image quality phantom, allowing for testing against the QIBA SLN profile.^25^

The aims of this study were 2-fold; firstly, to use readily available low dose LCS protocols, to evaluate radiation doses for 3 phantom sizes and objectively assess image quality using a range of reconstruction kernels and, secondly, to determine which kernels can be used to generate images that meet the QIBA SLN profile. This collated information could then act as a guide to the development of optimized LCS protocols across a range of contemporary scanners.

## Methods

Suggested LCS protocols for range of scanners were obtained from the AAPM protocol library^27^ and via contact with scanner manufacturers where necessary. These protocols were added to scanners in the local region. Details of the scanners that were included in the study are provided in Table 1. Two Canon Prime SP scanners and 2 Siemens go.All systems were tested to allow an assessment of the inter-scanner variation of image quality performance. All the scan protocols made use of the manufacturer’s automatic exposure control system. For most scanners that were included in the study, assessments were made of both dose and image quality. All scanners were subject to suitable quality assurance testing based on current UK guidance.

**Table 1:** List of scanners assessed as part of this study and the suggested reconstruction kernel(s).

Manufacturer	Model	Dose/image quality assessed?	Suggested reconstruction kernel(s)
Canon	ONE (Genesis)	Y/Y	Not specified
	Prime SP (×2)	Y/Y	Not specified
FujiFilm	Scenaria View	Y/Y	IPV—H lung LV2
GE	BrightSpeed 16	Y/Y	Lung or bone
	Revolution Evo	Y/Y	Lung or bone
	Revolution HD	Y/Y	Lung or bone
	VCT	N/Y^a^	Lung or bone
Philips	Incisive	Y/Y	Not specified
	Ingenuity	Y/Y	YA
	IQon	Y/Y	YA
Siemens	Definition AS+	Y/Y	I50/B31
	Definition Edge	Y/Y	I50/B31
	Emotion 16	Y/Y	B41s
	Somatom Drive	Y/Y	Bf37/Br59
	Somatom Force	Y/Y	Br40/Br64
	Somatom go.All (×2)	Y/Y	Br60
	Somatom X.cite	Y/Y	Br40

a Image quality was assessed shortly before the removal of this scanner from clinical use—we were unable to make an assessment of the dose performance at this time.

### Dose assessment

**Figure 1:**
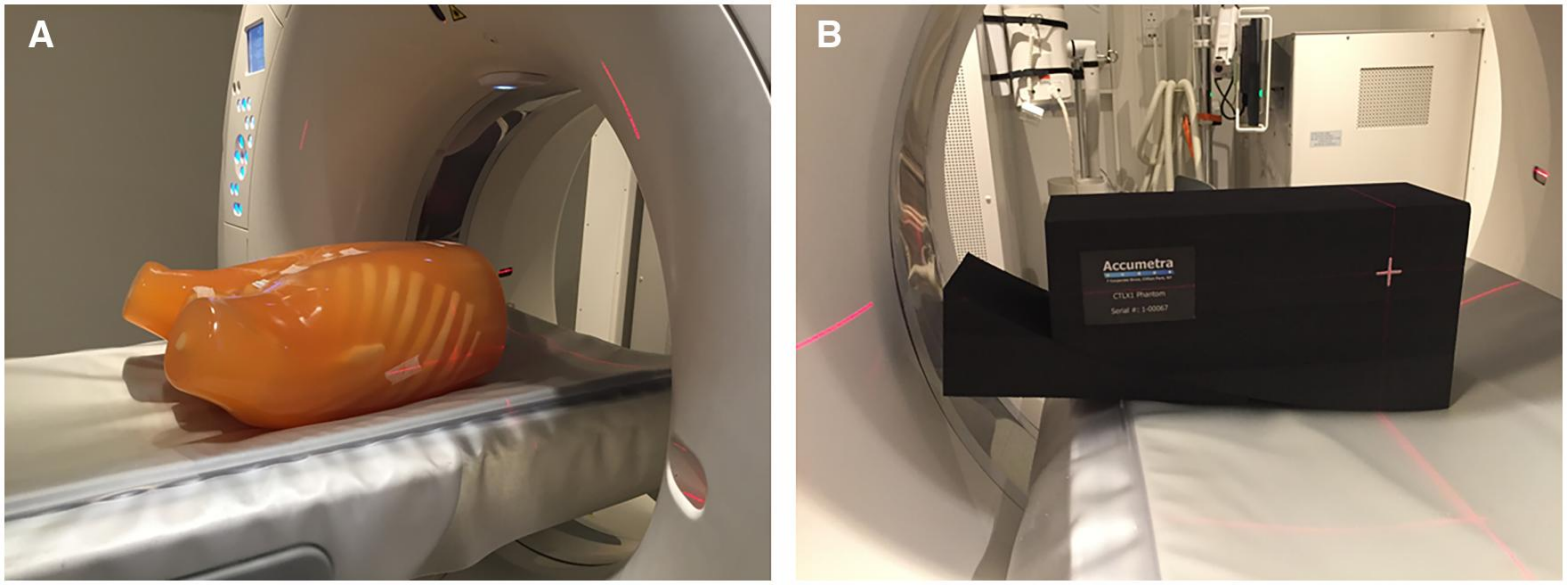
(A) The Lungman phantom and (B) the CTLX1 phantom used in this study.

For each scanner, the Kyoto Kagaku Lungman phantom was positioned isocentrically within the CT bore (Figure 1A), and the recommended scan projection radiographs (SPRs) were performed. A helical acquisition extending from lung apices to bases was performed, and the scanner displayed volume CT dose index (CTDIvol), and dose length product (DLP) were recorded. This process was repeated for 2 further phantom sizes by adding body plates to the posterior and anterior of the Lungman phantom. The water equivalent diameter (WED) of the 3 phantom sizes was calculated using the AutoWED software http://ctdose-iqurad.med.uoc.gr/autowed/, which calculates WED according to the AAPM TG220 methodology.^29^ Both CTDIvol and DLP were plotted as a function of WED to demonstrate the dose variation with phantom attenuation. The doses were compared with recommended levels for standard sized patients from the UK Targeted Lung Health Check (TLHC),^10^ AAPM,^27^ American College of Radiology (ACR),^22^ and the European Society of Thoracic Imaging (ESTI).^23^

Iball et al.^30^ previously identified an exponential relationship between CTDIvol and participant weight for lung screening scans on a Canon Prime SP scanner. Based on the CTDIvol values that were obtained on the same scanner in this study, we calculated the approximate patient weight that corresponded to the measured WED for the 3 phantom sizes.

### Image quality

The Accumetra CTLX1 phantom was used to objectively assess the image quality against the 6 metrics specified in the QIBA SLN profile.^25^ As for the dose measurements, this phantom was positioned isocentrically within the scanner (Figure 1B) and the appropriate SPRs were undertaken, following which a helical acquisition of the entire phantom was performed. Thin slice images of ≤1.25 mm were reconstructed for a range of reconstruction kernels, including that specified in the AAPM suggested protocol^27^ for the reconstruction specified as “lung.” The reconstruction kernels that were used were selected to cover a range from smooth to sharp. The QIBA SLN profile recommends that a medium-smooth to medium-sharp kernel is required for the highest objective image quality without edge enhancement.^25^ The CTLX1 images were uploaded to the Accumetra website (https://iservices.accumetra.com/CTLX1report.php) for analysis. The methods used to assess the CTLX1 phantom images are described in detail in the QIBA SLN profile.^25^ The CTLX1 phantom contains details at 0, 100, and 200 mm from the isocenter, enabling image quality to be assessed at 3 points across the imaged field and also interpolated to 160 mm from the isocenter. For each scanner, the measured data were used to determine whether the QIBA SLN criteria were met on the suggested kernel, and to establish the range of kernels that could be used to meet these criteria.

To assess the uncertainty in the measurements 5 scans were undertaken on the Siemens Somatom Force scanner, with the phantom removed from the scanner and repositioned for each acquisition. The standard deviation of the results was used to represent the measurement uncertainty.

Full details of the scan protocols used in the study are shown in the Supplementary Material.

## Results

**Figure 2:**
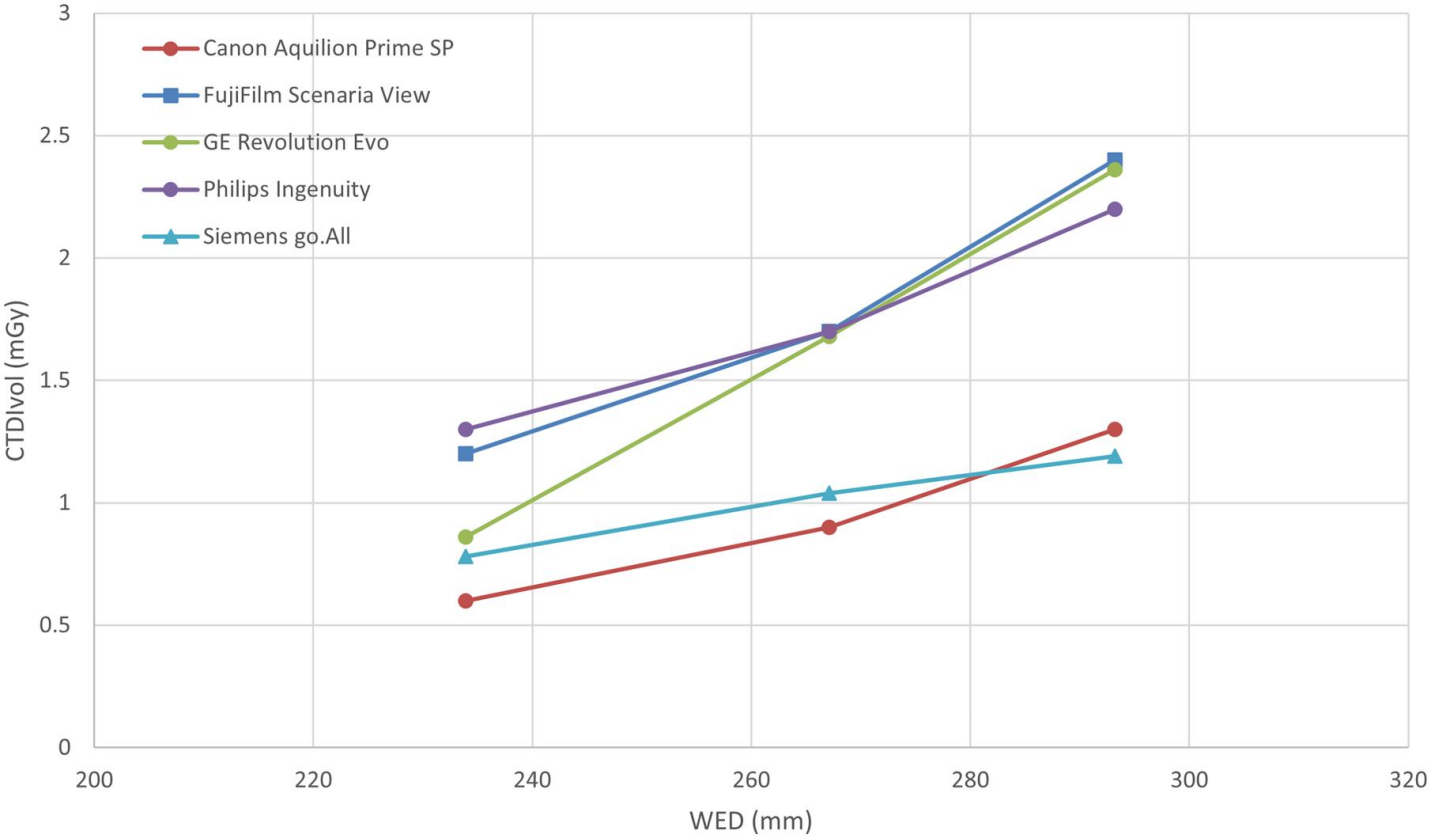
Showing the variation in CTDIvol for one scanner from each of the 5 manufacturers.

The CTDIvol and DLP values for the 3 phantom sizes are shown in Table 2. Doses varied between scanners by a factor of 7 for the smallest and 6 for the largest phantom size, respectively. The CTDIvol values for a selection of scanners are shown in Figure 2. DLP values showed the same trends as those shown for CTDIvol due to the fixed scan length that was used.

**Table 2:** Resulting values of CTDIvol (mGy) and DLP (mGycm) for the scans of the 3 chest phantom sizes on each of the scanner models.

		Canon		FujiFilm	GE			Philips			Siemens						
		Prime SP	ONE genesis	Scenaria View	BrightSpeed 16	Revolution HD	Revolution Evo	Ingenuity	IQon	Incisive	Definition AS+	Somatom Drive	Definition Edge	Emotion 16	Somatom Force	Somatom go.All	Somatom X.cite
CTDIvol (mGy)	Lungman	0.60	0.90	1.20	2.10	0.97	0.86	1.30	1.30	1.44	1.47	0.39	1.49	1.38	0.31	0.78	0.81
	Lungman + 1 cover	0.90	1.40	1.70	2.20	1.67	1.67	1.70	1.70	1.96	2.07	0.51	1.96	1.77	0.41	1.04	1.02
	Lungman + 2 covers	1.30	2.10	2.40	3.05	2.50	2.36	2.20	2.00	2.30	2.38	0.61	2.22	1.98	0.51	1.19	1.15
DLP (mGycm)	Lungman	19.7	29.6	39.4	69.0	31.9	28.3	42.7	42.7	47.3	48.3	12.8	49.0	45.3	10.2	25.6	26.6
	Lungman + 1 cover	29.7	46.2	56.1	72.6	55.1	55.5	56.1	56.1	64.7	68.3	16.8	64.7	58.4	13.5	34.3	33.7
	Lungman + 2 covers	42.9	69.4	79.3	100.8	82.6	78.0	72.3	66.1	76.0	78.6	20.2	73.3	65.4	16.9	39.3	38.0

Within the following figures, data are shown for 1 scanner model from each manufacturer. The Canon Prime SP and Siemens go.All were chosen as these are the scanners most frequently used for LCS services on mobile trailers within the UK. The scanners that are shown for FujiFilm, GE, and Philips were chosen as having broadly similar technical specifications to the Canon and Siemens systems.

The approximate weights of the 3 phantom sizes are shown in Table 3 which demonstrates that the middle phantom size is approximately 70 kg equivalent.

**Table 3:** Showing the calculated equivalent weight for the 3 phantom sizes.

Phantom size	WED (mm)	CTDIvol (mGy) on Canon Prime SP	Equivalent weight (kg)
Lungman	234	0.6	49.0
Lungman +	267	0.9	68.7
Lungman ++	293	1.3	86.7

The CTDIvol values, and calculated effective dose, for the middle phantom size, were compared with the AAPM/ACR, ESTI and TLHC standards. When using the recommended scan protocols, all scanners achieved the 3 mGy AAPM/ACR and 2 mSv TLHC standards, but only 2 met the 0.8 mGy ESTI criteria. Three further scanners met the ESTI 1.6 mGy threshold for >80 kg patients but did not achieve the dose values specified for the lower weight bands. Effective dose was calculated using a 0.027 mSv/mGycm conversion factor^31^ which is based on a scan covering lung apices to bases of the ICRP 110 computational phantoms^32^ using the ICRP 103 tissue weighting factors.^33^

Table 4 shows the results of the image quality assessment, identifying whether the QIBA SLN profile was met using the suggested reconstruction kernel(s). Six of the 17 scanners tested achieved the QIBA SLN profile criteria using the recommended reconstruction settings. This demonstrates that 11 scanners failed in terms of edge enhancement, 12 based on 3D resolution aspect ratio and 1 on each of 3D resolution, spatial warping, HU bias, and image noise.

**Table 4:** Performance against the QIBA SLN profile for the suggested reconstruction kernel(s), and the kernels achieving the QIBA SLN profile.

Manufacturer	Model	Kernel(s) suggested in AAPM protocols	QIBA SLN profile met with suggested kernel?	Reasons for failure to meet QIBA SLN profile	List of kernels meeting QIBA SLN profile
Canon	ONE (Genesis)^a^	FC51^b^	No	3DAR	FC5, FC7, FC15
	Prime SP 1^a^	FC51^b^	No	EE, 3DAR	FC5, FC7, FC9
	Prime SP 2^a^	FC51^b^	No	EE, 3DAR	AiCE Body Sharp
FujiFilm	Scenaria View	IPV—H Lung LV2	No	EE, 3Dres, 3DAR	Abdo IPA Sharp 2, Abdo IPA Sharp 3
GE	BrightSpeed 16	Lung	No	EE, SW, 3DAR	None identified
		Bone	No	EE, SW, 3DAR	
	Revolution Evo	Lung	No	EE, 3DAR	Standard, Detail, Edge
		Bone	No	3DAR	
	Revolution HD	Lung	No	EE, 3DAR	Standard, Detail, Chest, Edge
		Bone	No	EE, 3DAR	
	VCT	Lung	No	EE	Standard
		Bone	No	EE, 3DAR	
Philips	Incisive^c^	A^b^	Yes	N/A	Lung A, Lung B, Lung C, Lung YA, Lung YB, Lung PI Sharp, Lung PI Smooth, Soft Tissue PI Sharp, Soft Tissue PI Smooth, Soft Tissue PI Standard
		YA^b^	Yes	N/A	
	Ingenuity	YA	Yes	N/A	A, B, C, YA, iMR Routine
	IQon	YA	Yes	N/A	A, B, C, YA, iMR Routine, iMR Soft Tissue
Siemens	Definition AS+	I50f	No	EE, 3DAR	I30f, I40f, I41f
	Definition Edge	B31s	Yes	N/A	I26f, I30f, I31f, I40f, I41f
	Emotion 16	B41s	Yes	N/A	B10s, B20s, B30s, B31s, B40s, B41s
		B70s	No	3DAR, Noise	
	Somatom Drive	Bf37	Yes	N/A	Bf37, Bf39, Bf42
		Br59	Not tested	N/A	
	Somatom Force	Br40	No	EE	Bf40
		Br64	No	EE, 3DAR, HU	
	Somatom go.All	Br60	No	EE, 3DAR, Noise	Br40, Br44
	X.cite	Br40	No	EE	None identified

Abbreviations: 3DAR = 3-dimensional resolution aspect ratio; 3Dres = 3-dimensional resolution; EE = edge enhancement; HU = Hounsfield Unit bias; SW = spatial warping.

a The AAPM protocols specify the ^SURE^IQ as Lung Standard Axial, but do not specify the reconstruction kernel itself.

b Reconstruction kernel suggested by manufacturer.

c The Philips Incisive scanner is not included in the AAPM protocol list.

All failures were correctable through selection of an alternative reconstruction kernel, with the exception of the spatial warping failure on the GE BrightSpeed 16 and the edge enhancement failure on the Siemens X.cite. Table 4 also presents a list of kernels that could be used to meet the QIBA SLN profile on each of the scanners.

**Figure 3:**
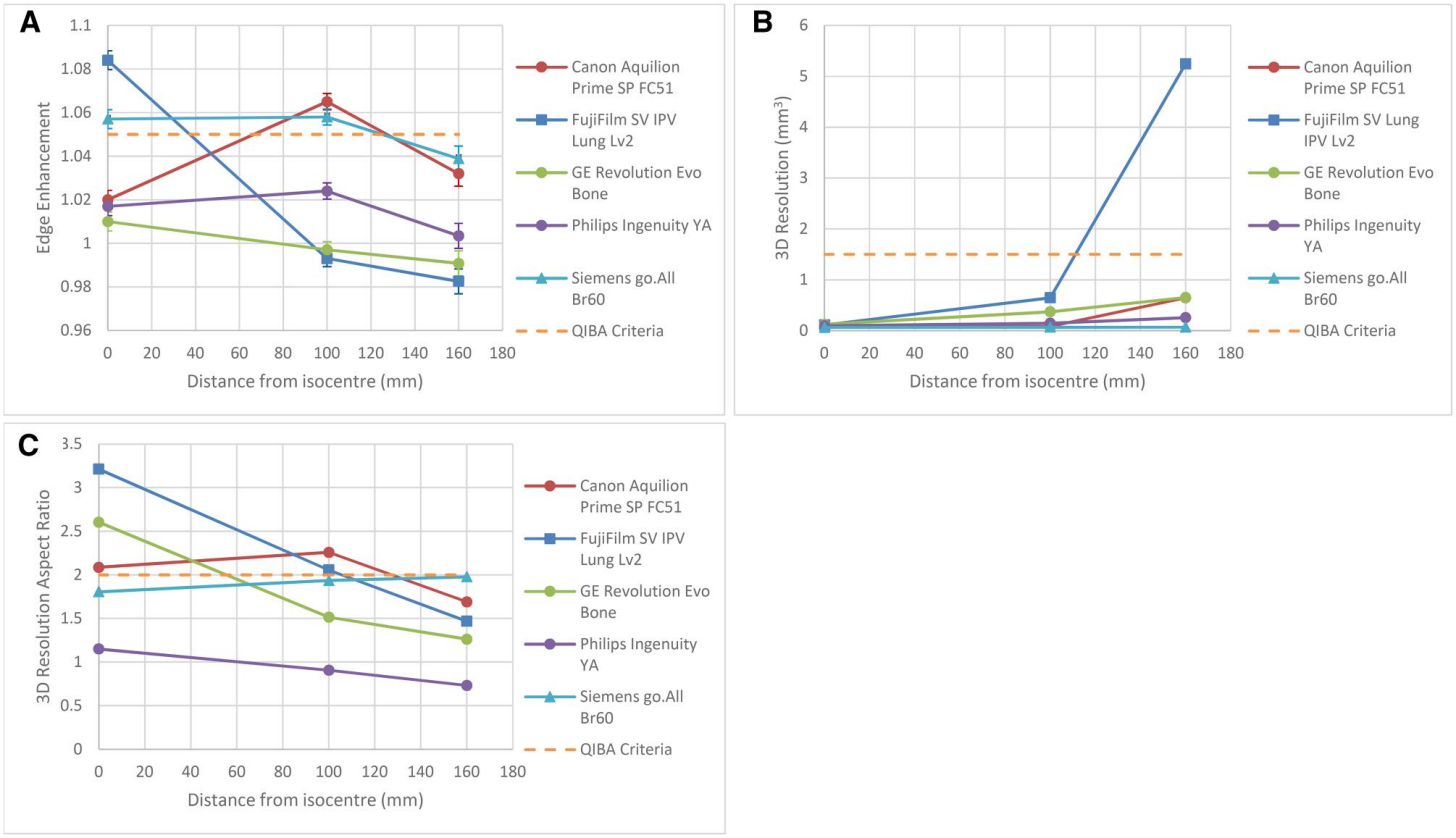
Image quality measurements on suggested reconstruction kernels for 1 scanner from each manufacturer: (A) edge enhancement, (B) 3D resolution, (C) 3D resolution aspect ratio. Error bars represent 1 standard deviation—these are smaller than the data points in (B and C).

**Figure 4:**
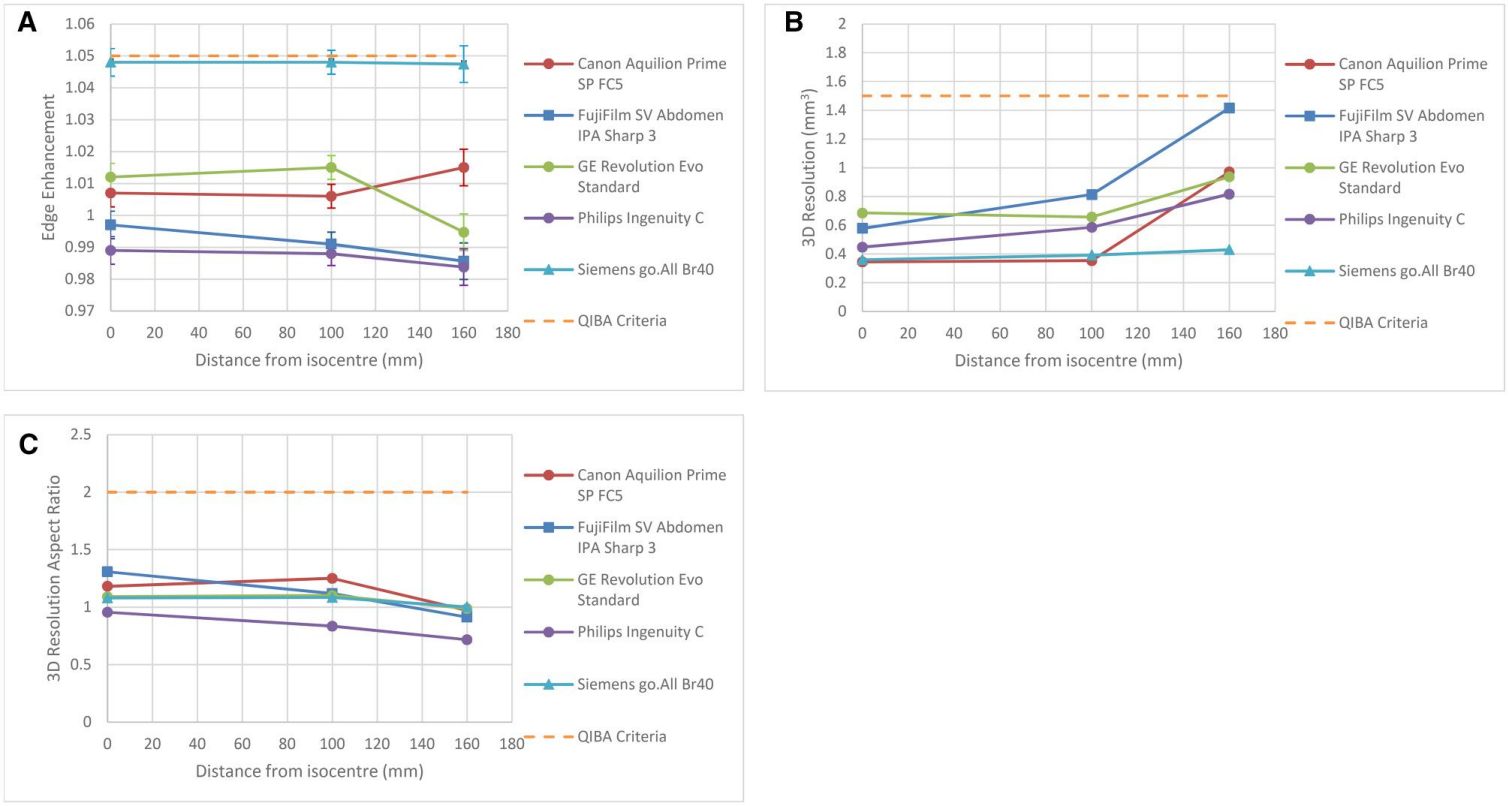
Image quality measurements for a reconstruction kernel that met the QIBA SLN profile criteria for one scanner from each manufacturer: (A) edge enhancement, (B) 3D resolution, (C) 3D resolution aspect ratio. Error bars represent 1 standard deviation—these are smaller than the data points in (B and C).

Figure 3 shows the image edge enhancement, 3D resolution, and 3D resolution aspect ratio results for the suggested reconstruction kernel for 1 scanner from each manufacturer. Edge enhancement and 3D resolution aspect ratio were chosen as these were the metrics for which the greatest number of failures were recorded. The 3D resolution is also presented as there are notable differences in the results between manufacturers. The results in Figure 4 are for the same scanners but using a reconstruction kernel that met the QIBA SLN profile criteria.

**Figure 5:**
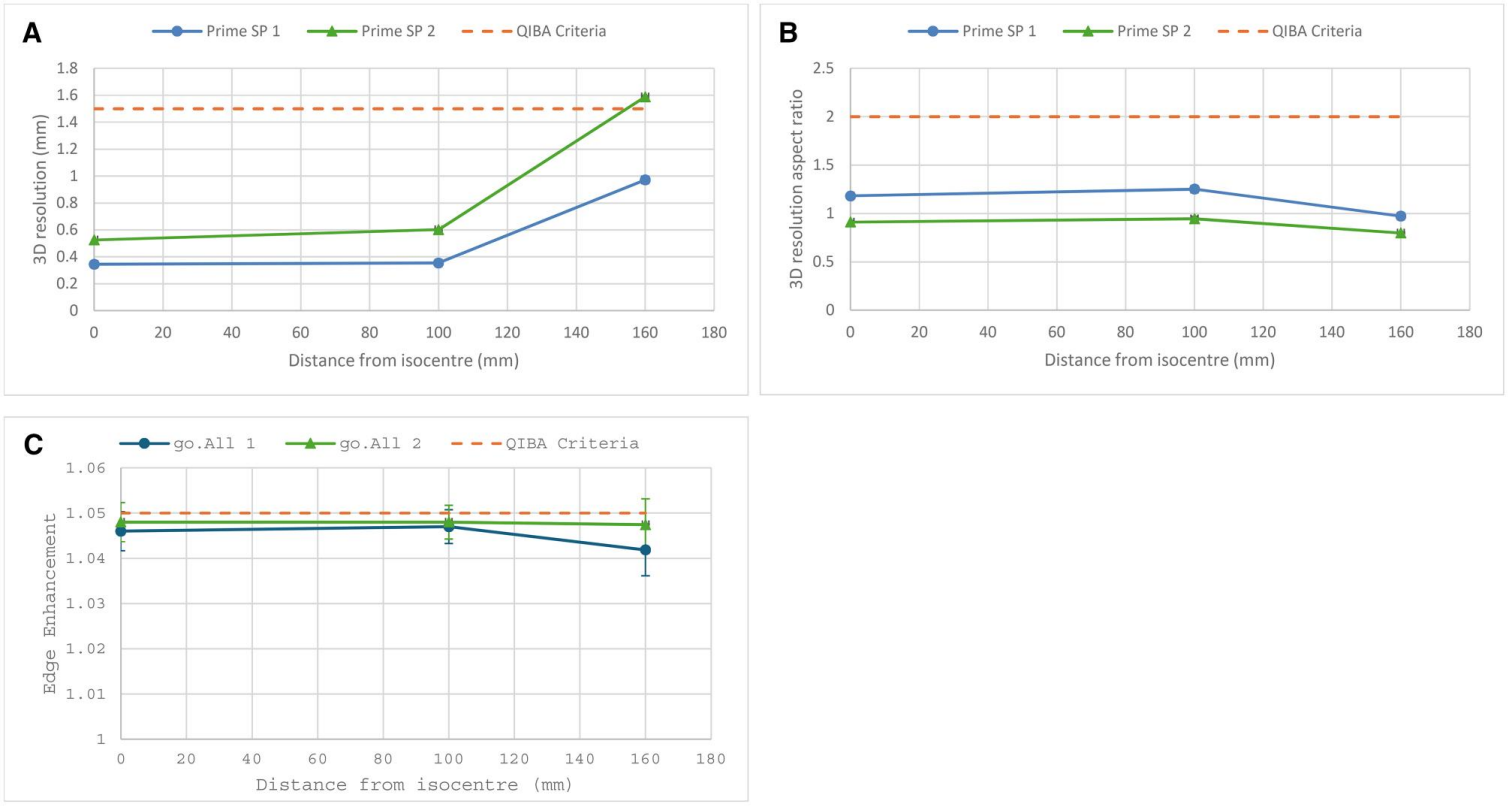
Comparative image quality data for 2 Canon Prime SP scanners: (A) 3D resolution, (B) 3D resolution aspect ratio, and 2 Siemens go.All scanners: (C) edge enhancement. Error bars represent one standard deviation—these are smaller than the data points in (A and B).

A selection of the comparative image quality results for the 2 Canon Prime SP and 2 Siemens go.All scanners are shown in Figure 5A-C. The results were highly comparable between the Siemens scanners with only small deviations in edge enhancement towards the periphery of the imaged field, but these were within the uncertainties in the measurements. For the Canon scanners there was notable difference in the 3D resolution which resulted in Prime SP2 failing the QIBA SLN profile criteria at the periphery of the field.

## Discussion

Our results have demonstrated notable variations in radiation dose and image quality performance across a range of CT scanners for CT lung cancer screening protocols.

Since each of the 19 scanners tested, which were aged from 1 to 10 years, were able to meet both the AAPM/ACR^22^ and TLHC dose requirements^10^ it may be necessary to review these dose recommendations to better reflect the capabilities of modern CT systems and ensure that radiation doses are kept as low as reasonably practicable.

The 2 scanners that were able to meet the ESTI dose requirements^23^ when using a single scan protocol were dual-source CT systems (Siemens Somatom Force and Drive) which are amongst the highest specification systems offered by that manufacturer. As such, these are relatively high-cost systems which may prohibit their broad adoption within a geographic LCS program. It is likely that other CT systems within the study would be able to meet the 3 ESTI dose levels through adaptation of the AEC settings or use of weight-based scan protocols but investigating this was outside the scope of this study. The recommended ESTI dose values are half of those that were used in the NELSON study^34^ which was conducted between 2003 and 2009. Although improvements in CT technology should enable modern scanners to operate at lower doses, it is not clear what evidence supports the assumption that the ESTI dose values accurately reflect the performance of current scanners.

The lowest doses identified within the study were for 4 Siemens scanners (Force, Drive, go.All, and X.cite) and the Canon Prime SP. The 4 Siemens scanners utilized additional tin filtration to remove low energy X-ray photons from the spectrum (Figure 2). CTDIvol values for these 3 scanners were between 46% and 79% lower than those on the Siemens scanners without tin filtration. Whilst other hardware and software features may have also contributed to this reduction in dose, these results demonstrate the capability of the tin filter for reducing dose in LCS scans. Canon have incorporated a silver filter into some of their CT models for this same purpose, although this was not available on the Canon scanners used in this study. A recent study by Golbus et al.^35^ identified that for chest scans undertaken with the silver filter and an artificial intelligence reconstruction process (AiCE) doses were reduced by 85.5% compared with scans without the silver filter reconstructed with an iterative reconstruction algorithm (AIDR 3D). Whilst it is unclear what percentage of the dose reduction was directly attributable to the silver filter, their findings are in broad agreement with those of this study: that additional beam filtration can lead to notably reduced doses for LCS scans.

Our results have also demonstrated differences in the performance of the different manufacturers Automatic Exposure Control (AEC) systems as observed from the gradients of the plots of CTDIvol against water equivalent diameter. These results echo those from earlier studies that showed AEC system performance differed according to the manufacturer’s design philosophy with Canon & GE adopting a constant-noise approach and Philips & Siemens instead opting for an adequate-noise system.^36^^,^^37^ Knowledge of how the AEC system adjusts dose according to patient habitus is important information for users to be aware of when developing LCS protocols, especially where there is a desire to comply with weight-based dose values, such as those provided by ESTI.^23^ Care must be taken to ensure that for larger patients, dose levels are adjusted sufficiently to ensure that accurate nodule volumetry can still be achieved in these more challenging subjects.

With careful selection of reconstruction kernel, all the scanners were able to produce images that met the 6 criteria within the QIBA SLN profile, except for the GE BrightSpeed 16 and Siemens X.cite scanners. The results for the GE BrightSpeed 16 are in agreement with those obtained by other users of the CTLX1 phantom, whilst other users testing Siemens X.cite scanners have identified protocols which met all criteria in the QIBA SLN profile (R. Avila, personal communication, February 2025). Further work is required to identify whether additional optimization steps would cause the X.cite scanner to meet the QIBA SLN profile, and what the clinical effect of not meeting the profile is. Both of these avenues of inquiry are outside the scope of this current study.

Our results demonstrate that on most scanners, images produced using traditional “lung” reconstruction kernels were unable to meet the QIBA SLN profile criteria. The most common reasons for these failures were excessive edge enhancement and a mismatch in the in-plane and longitudinal spatial resolution (3D aspect ratio). To achieve the highest level of objective image quality a soft tissue reconstruction kernel was required on most scanners. This is as recommended within the QIBA SLN profile, which states: “Reconstruction Kernel is recommended to be a medium smooth to medium sharp kernel that provides the highest resolution available without edge enhancement.”

For 2 Canon scanners the FC5 and FC7 kernels enabled the QIBA SLN profile to be met and these should be considered for adoption on other Canon scanners for LCS scans. For the Canon Prime SP scanner equipped with AiCE, the FC5 kernel images did not meet the QIBA SLN profile criteria, but this was achieved through use of the AiCE Body Sharp algorithm. For the GE systems that were tested, the QIBA SLN profile was achieved with the “Standard” kernel on all 3 scanners and with “Detail” and “Edge” kernels on 2 scanners. It should be noted that “Edge” is a relatively sharp kernel and does not fit with the QIBA SLN profile suggestion of “medium-smooth” to “medium-sharp.” As such we propose that the “Standard” kernel could be utilized on GE scanners. Only one FujiFilm scanner was included in the study and the kernels that met the QIBA SLN profile criteria were “Abdomen IPA Sharp 2” and “Abdomen IPA Sharp 3.”

The 3 Philips scanners were able to achieve the QIBA SLN profile requirements on 22 of the 27 kernels that were tested, with failures only encountered for the very sharpest kernels (YB, IMR Sharp & Lung PI Standard). These results imply that the Philips kernels generally do not apply high levels of edge enhancement, other than on these very sharp kernels. A 768 × 768 image matrix was used on the Philips scanners whereas the standard 512 × 512 matrix was used on all other scanners. As noted by both Euler et al.^38^ and Hata et al.,^39^ use of a larger matrix size can lead to improved spatial resolution in chest CT scans, *albeit* at the expense of increased image noise. It should be noted however, that it is the combination of the inherent resolution of the CT detection system, frequency properties of the reconstruction kernel and the matrix size which determine the image resolution, and simply adjusting the matrix size may not increase spatial resolution if the kernel frequency properties or detection system itself are the limiting factors. For the Philips scanners the larger matrix size enabled higher in-plane spatial resolution, and this, combined with the 50% overlapping image slices, resulted in low 3D resolution values across the imaged field, and a 3D resolution aspect ratio of approximately unity. It is noted that it is now becoming more common to utilize 768 × 768 or 1024 × 1024 matrices in chest CT imaging^38^^,^^40^ and as such a general adoption of higher matrix sizes should reduce the number of failures on the resolution aspects of the QIBA SLN profile. A direct result of the reduced voxel sizes associated with the 768 × 768 matrix is the increase in noise values, with the Philips scanners generally yielding higher noise values than on broadly equivalent kernels from other manufacturers. For the Philips scanners, the range of reconstruction kernels that can be used to meet the QIBA SLN profile is therefore large and would allow the clinical site to operate a high degree of user preference with regards this.

The Siemens scanners showed a model dependent variation in the availability of reconstruction kernel options, which may be because of differences in detector construction, detector element size, and system geometry. As such it is not possible to give strong guidance as to which reconstruction kernels will enable the QIBA SLN profile to be met on any given Siemens system. This demonstrates that compliance testing needs to be undertaken on each scanner rather than at the level of a scanner model. Kernels in the number range 37-40 were most frequently able to meet the criteria, but on some scanners, these were body regular (Br) kernels whilst on other scanners body fine noise (Bf) kernels were needed. Selecting kernels in the range Br37-Br40, or Bf37-Bf40 will likely enable the scanner to meet the QIBA SLN profile. However, the Siemens X.cite scanner included in this study did not achieve the QIBA SLN criteria on any available kernel. Discussion with Accumetra identified that other CTLX1 users have met the QIBA SLN profile with this same model of scanner using the Br40 reconstruction kernel (R. Avila, personal communication, February 2025).

The comparative data from the 2 Siemens go.All systems demonstrated that edge enhancement, more than any other metric, appeared to be subject to inter-scanner variation (Figure 5C). Since the X.cite we tested failed this criterion, this inter-scanner variation may explain why other X.cite scanners have met the edge enhancement criteria using the same reconstruction parameters. It should be noted that even with consideration of the measurement uncertainties the X.cite still failed to meet the edge enhancement criterion. Similarly differences in the 3D resolution results for 2 Canon Prime SPs using the identical scan protocol resulted in 1 scanner achieving, and 1 failing the QIBA SLN profile criteria. These results imply that there could be clinically significant differences between the performance of scanners of the same model, and this requires further investigation with a larger number of identical scanners.

The results of the image quality measurements allow us to make strong indications of the reconstruction kernels that could be used to meet the QIBA SLN profile on Canon, FujiFilm, GE and Philips systems, and to give a broad indication of the kernels that may deliver this level of performance on Siemens scanners. However, given the variations that have been observed between similar scanners, and also across different scanners from the same manufacturer, we recommend that the image quality performance of each scanner that is used for LCS is evaluated to identify and account for any performance variations resulting from differences in scanner set-up, calibration, software version and scan geometry. Indeed, should a LCS program adopt the QIBA SLN profile image quality criteria, the LCS scan protocols on each individual scanner would need to be assessed. The findings of this study indicate that it is highly likely that appropriate scan protocol adjustments would result in the QIBA SLN profile criteria being met.

It is also worth noting that for all scanners there were observed variations in spatial resolution, image noise, or both towards the periphery of field (Figures 3A-C and 4A-C). These variations occur as a result of the X-ray paths being more densely distributed towards the center of the scan field of view compared with the periphery of the field which results in increased sampling towards the center. In addition, differences in how the manufacturers are handling voxel data across the imaged field further contributes to the variations in image quality metrics across the field of view. These variations in imaging performance could have implications for the accuracy of nodule volumetry towards the periphery of the lungs.

As a result of this study, we recommend that information regarding expected dose values for a range of water equivalent diameters, and which reconstruction kernels allow the scanner’s performance to meet the QIBA SLN profile, should be incorporated into a standard LCS protocol repository such as that provided by AAPM. This would allow current and prospective LCS providers to understand how the scan protocols perform and where further optimization steps may be required. Further work is needed to assess dose and image quality in this way for other modern scanners, and to keep any such protocol repository updated as new scanner models become available.

In addition, we recommend that LCS programs should invest in the most technologically advanced scanners to reduce radiation doses as far as is practicable whilst ensuring that high-quality images are generated for automated volumetry purposes. We note that most scanners installed on mobile trailers are not the highest specification systems available, and as such this recommendation is at odds with current practice. Further work is needed to assess the protocol adjustments that would be required to enable scanners to meet the ESTI dose levels^23^ and to evaluate what effect these changes would have on image quality, specifically image noise.

There are limitations with this study. Firstly, although we were able to include data from 5 different CT manufacturers, small numbers of scanner models were included for some manufacturers, and not all scanners available for LCS within the UK were tested. However, having tested 17 different scanner models we are confident in the broad applicability of our findings.

The image quality phantom used in the study, Accumetra CTLX1, is not patient equivalent and does not therefore allow the evaluation of image quality metrics at levels of attenuation experienced in clinical LCS scans. Accumetra now manufacture a variable size phantom containing the same test details (CTLX2) which enables the assessment of image quality under patient equivalent attenuation conditions. There would be value in undertaking further assessments of image quality with this phantom.

The study does not address the clinical effect that would result from the failure to meet the QIBA SLN profile requirements. This should be the focus of future studies.

We were not able to provide protocol settings that would deliver the optimized balance of dose and image quality for each scanner model as there are many additional factors, such as image slice thickness and overlap, matrix size, iterative reconstruction level and use of deep learning-based reconstruction that could be adapted to improve image quality performance, in addition to parameters such as tube voltage, helical pitch and AEC settings that could be adjusted to further reduce doses. This level of individual scanner optimization, as demonstrated in a recent study,^41^ is of value to the LCS community but is beyond the scope of this study.

In conclusion, our study has shown that it is unlikely that recommended LCS scan protocols are optimized in terms of their ability to deliver consistently accurate volumetric analysis of lung nodules at low dose. However, specialized test equipment can be used to assess performance against objective image quality criteria, enabling careful adjustment of scanner protocols to ensure that these criteria are met. Whilst all of the scanners tested were able to meet recommendations regarding radiation dose, the use of additional filtration enables further dose reduction. Our study demonstrated the value of assessing scan protocol performance on every CT scanner undertaking LCS examinations. With these measures in place, it is likely that the majority of modern scanners can be configured to deliver accurate nodule volumetry for LCS examinations at low dose.

## Supplementary Material

tqag066_Supplementary_Data
