# Supplementary material for: Towards optimized CT lung cancer screening scan protocols
Source: Br J Radiol. 2026 Mar 26;99(1183):1338–48. doi: 10.1093/bjr/tqag066 (PMC13274652; doi:10.1093/bjr/tqag066)
Supplement: tqag066_Supplementary_Data [file tqag066_supplementary_data.docx]

| Manufacturer | Canon | | | | FujiFilm | | | GE | | | | | | | | | |
| --- | --- | --- | --- | --- | --- | --- | --- | --- | --- | --- | --- | --- | --- | --- | --- | --- | --- |
| Model | Prime SP | | ONE (Genesis) | | Scenaria View | | | BrightSpeed 16 | | Revolution Evo | | | Revolution HD | | VCT | | |
| Rotation time (s) | 0.35 | | 0.35 | | 0.5 | | | 0.5 | | 0.5 | | | 0.5 | | 0.5 | | |
| Detector configuration (mm) | 80 x 0.5 | | 80 x 0.5 | | 64 x 0.625 | | | 16 x 1.25 | | 64 x 0.625 | | | 64 x 0.625 | | 64 x 0.625 | | |
| Pitch | 0.813 | | 0.813 | | 0.83 | | | 1.375 | | 1.375 | | | 1.375 | | 0.984 | | |
| Tube voltage (kV) | 120 | | 120 | | 120 | | | 120 | | 120 | | | 120 | | 120 | | |
| Automatic exposure control (AEC) used? | Yes (SURE Exposure) | | Yes (SURE Exposure) | | Yes (Intelli EC) | | | Yes (AutomA & SmartmA) | | Yes (AutomA & SmartmA) | | | Yes (AutomA & SmartmA) | | Yes (AutomA & SmartmA) | | |
| AEC settings | Image thickness 5mm  sd = 25  mA range: 20-120  SureIQ: FC13 | | Image thickness 5mm  sd = 25  mA range: 20-120  SureIQ: FC13 | | sd = 25  mA range: 20-120  Intelli EC Plus | | | NI = 32  40-130mA | | NI = 29.5  20-290mA | | | NI = 34  20-290mA | | NI = 32  30-110mA | | |
| Image slice thickness / interval (mm) | 1 / 1 | | 1 / 0.8 | | 1 / 0.5 | | | 1.25 / 0.625 | | 1.25 / 0.625 | | | 1.25 / 0.625 | | 1.25 / 0.625 | | |
| Image matrix | 512 | | 512 | | 512 | | | 512 | | 512 | | | 512 | | 512 | | |
| Reconstruction method | AIDR 3D / AiCE | | AIDR 3D | | Intelli IPA / Intelli IPV | | | ASIR | | ASIR-V | | | ASIR-V | | ASIR | | |
| Iterative reconstruction settings | Standard | | Standard | | Level 3 | | | 30 | | 30 | | | 30 | | 30 | | |
| Reconstruction kernels | Prime 1: FC5, FC7, FC9, FC15, FC51  Prime 2: FC5, AiCE Lung, AiCE Body Sharp | | FC5, FC7, FC9, FC15, FC51 | | Abdo IPA Sharp 1-3,  Abdo IPA Smooth 1-2, Abdo IPA Std, Abdo IPV Std, Lung IPA Sharp 1-2, Lung IPA Smooth 1-2, Lung IPA Std, Lung IPV Std | | | Standard, Bone | | Soft, Standard, Lung, Detail, Chest, Edge, Edge+ Bone, Bone+ | | | Soft, Standard, Lung, Detail, Chest, Edge, Edge+ Bone, Bone+ | | Standard, Lung, Bone | | |
| Manufacturer | Philips | | | | | Siemens | | | | | | | | | | |  |
| Model | Ingenuity | IQon | | Incisive | | Definition AS+ | Somatom Drive | | Definition Edge | | Emotion 16 | Somatom Force | | Somatom go.All | | Somatom X.cite |  |
| Rotation time (s) | 0.4 | 0.4 | | 0.4 | | 0.33 | 0.5 | | 0.33 | | 0.6 | 0.5 | | 0.33 | | 0.30 |  |
| Detector configuration (mm) | 64 x 0.625 | 64 x 0.625 | | 64 x 0.625 | | 128 x 0.6 | 128 x 0.6 | | 128 x 0.6 | | 16 x 0.6 | 192 x 0.6 | | 32 x 0.7 | | 64 x 0.6 |  |
| Pitch | 1.1 | 1.0 | | 1.2 | | 1.0 | 1.2 | | 1.0 | | 1.5 | 1.2 | | 0.8 | | 0.6 |  |
| Tube voltage (kV) | 120 | 120 | | 120 | | 120 | Sn100 | | 120 | | 110 | Sn100 | | Sn100 | | Sn100 |  |
| Automatic exposure control (AEC) used? | Yes (DoseRight) | | | | | Yes (CARE Dose 4D) | | | | | | | | | | |  |
| AEC settings | DRI = 6 | DRI = 5 | | DRI = 6 | | Q.Ref mAs = 25 | Q.Ref mAs = 81 | | Q.Ref mAs = 25 | | Q.Ref mAs = 20 | Q.Ref mAs = 101 | | Q.Ref mAs = 108 | | Q.Ref mAs = 114  CAREkV IQ level = 15 |  |
| Image slice thickness / interval (mm) | 1 / 0.5 | 1 / 0.5 | | 1 / 0.5 | | 1 / 0.7 | 1 / 0.7 | | 1 / 0.7 | | 1 / 0.7 | 1 / 0.7 | | 1 / 0.7 | | 1 / 0.7 |  |
| Image matrix | 768 | 768 | | 768 | | 512 | 512 | | 512 | | 512 | 512 | | 512 | | 512 |  |
| Reconstruction method | iDose & IMR | iDose & IMR | | iDose & PI | | SAFIRE | ADMIRE | | SAFIRE | | FBP | ADMIRE | | ADMIRE | | ADMIRE |  |
| Iterative reconstruction settings | iDose 5 &  IMR | iDose 5 &  IMR | | iDose 5 & PI | | Strength 2 | Strength 3 | | Strength 2 | | N/A | Strength 3 | | Strength 3 | | Strength 3 |  |
| Reconstruction kernels | A, B, C, YA, YB, IMR Routine, IMR Soft Tissue, IMR Sharp | A, B, C, YA, YB, IMR Routine, IMR Soft Tissue, IMR Sharp | | Lung A, B, C, YA, YB, Lung PI Sharp,  Lung PI Smooth, Lung PI Std,  ST PI Sharp, ST PI Smooth, ST PI Std | | I30f, I40f, I41f, I50f, I70f | Br32, Bf37, Bf39, Bv38, Bv40, Bf42 | | I26f, I30f, I31f, I40f, I41f, I44f, I50f, I70f | | B10s, B20s, B30s, B31s, B40s, B41s, B60s, B70s | Br32, Br36, Br40, Bf40, Br49, Bl57, Br64 | | Br36, Br40, Br44, Br48, Br56, Br60, Bl56 | | Br32, Br36, Br40, Br44 |  |
